# The influence of sex difference on behavior and adult hippocampal neurogenesis in C57BL/6 mice

**DOI:** 10.1038/s41598-023-44360-8

**Published:** 2023-10-12

**Authors:** Chi-Hui Tsao, Kuan-Yu Wu, Nicole Ching Su, Andrew Edwards, Guo-Jen Huang

**Affiliations:** 1 Graduate Institute of Biomedical Sciences, College of Medicine, Chang Gung University, Taoyuan, 333 Taiwan; 2 Department of Biomedical Sciences, College of Medicine, Chang Gung University, Taoyuan, 33302 Taiwan; 3 Department of Psychiatry, Dykebar Hospital, National Health Service Greater Glasgow and Clyde, Paisley, PA2 7DE Scotland; 4 Department of Neurology, Chang Gung Memorial Hospital-Linkou Medical Center, Taoyuan, 333 Taiwan; 5 Molecular Medicine Research Center, Chang Gung University, Taoyuan, 333 Taiwan

**Keywords:** Adult neurogenesis, Emotion, Learning and memory, Sensorimotor processing

## Abstract

Animal models have been used extensively in in vivo studies, especially within the biomedical field. Traditionally, single-sex studies, mostly males, are used to avoid any potential confounding variation caused by sex difference and the female estrous cycle. Historically, female animal subjects are believed to exhibit higher variability, and this could increase the statistical power needed to test a hypothesis. This study sets out to evaluate whether a sex difference does exist in mouse behavior, and whether female mice featured higher variability. We assessed the sensorimotor skills, anxiety-like behavior, depression-like behavior, and cognitive abilities of mice through a series of commonly used behavioral tests. Except for the stronger grip force and lower tactile sensory sensitivity detected in male mice, there was no significant difference between males and females in other tests. Furthermore, immunolabeling of neurogenesis markers suggested no significant difference between sexes in adult hippocampal neurogenesis. Within group variances were equivalent; females did not exhibit higher variability than males. However, the overall negative results could be due to the limitation of small sample size. In conclusion, our study provides evidence that sex difference in mice does not significantly influence these commonly used behavioral tests nor adult neurogenesis under basal conditions. We suggest that female mice could also be considered for test inclusion in future experiment design.

## Introduction

Animal models have been used in scientific research for over two thousand years, dating back to ancient Greece^1^. Among them, mice (Mus musculus) serve as one of the best model organisms for their genetic similarity to humans, ease of breeding, and well-established databases^2^. Nevertheless, sex difference has been observed in rodents, with males and females exhibiting different anatomical structures, physiology, and certain behaviors^3–5^. Historically, it has also been viewed that the estrous cycle increases variation in females relative to their male counterparts. Such divergence between sexes can be attributed to a combination of genetic, hormonal, and environmental factors.

For decades, cohorts of exclusively male mice have been utilized to avoid the confounding effects of the female estrous cycle and other potential variables which may interfere with experimental outcomes^6^. However, recent studies have challenged the use of a single sex in animal research. The underrepresentation of female subjects not only limits our understanding, but is also likely to lead to sex-biased results. In 2016, the National Institutes of Health (NIH) implemented a policy that requires the use of both sexes in vertebrate animal research, highlighting the importance of sexual effects. Therefore, the inclusion of female subjects is critical to improve the generalizability and to ensure both the validity and reliability of research findings. With the increasing attention, sex difference in animal studies has been an active issue in biological research. Understanding the impact of sex difference will be of great importance for both advancing our knowledge and application of experimental animals.

In our study, we aimed to examine potential sex differences in animal behavior, along with a comparison of variances in males and females. C57BL/6 mice were chosen in this study due to their lower anxiety compared to other inbred strains, which makes them better subjects for animal behavior assessment^7^. They are also one of the most widely used inbred strains and serves as a background for many transgenic lines in neurobiological research. We subjected 12 male and 12 female C57BL/6 mice to a series of behavioral tests to measure their sensorimotor skills, anxiety-like behavior, depression-like behavior, and cognitive abilities. Adult hippocampal neurogenesis was also investigated since a sex effect in rat adult neurogenesis had been demonstrated^8^. Our study provides an overview of sex differences in these commonly used behavioral assays and could serve as a reference for the mouse model used in scientific research.

## Results

### Males exhibited stronger grip force and lower tactile sensory sensitivity

**Figure 1:**
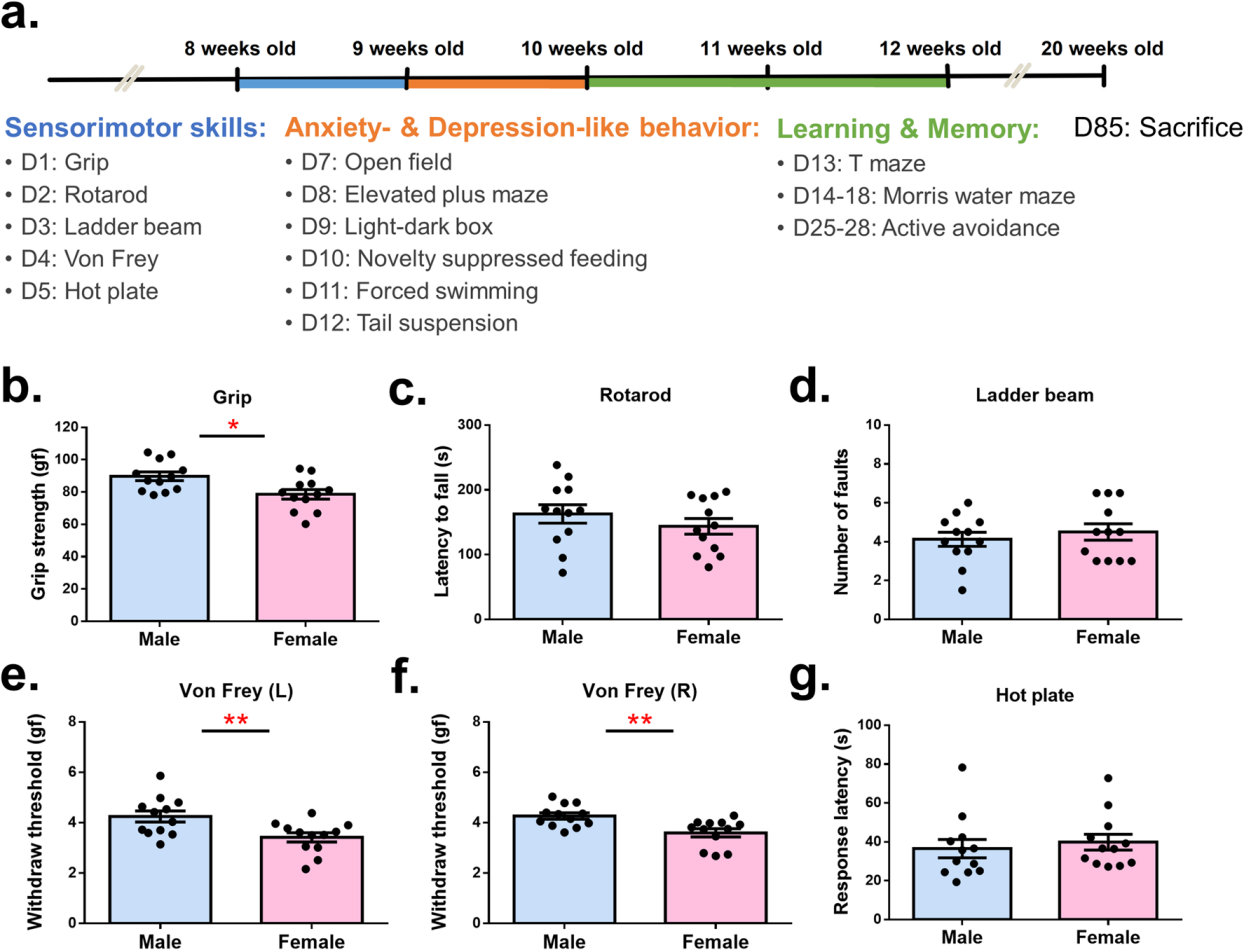
Males exhibited stronger grip force and lower tactile sensory sensitivity. (**a**) Timeline of the experimental design. D stands for the day after the assessment of behavior. (**b**) Male mice exhibited stronger grip force than females did. (**c**) No significant difference was observed between sexes in the latency to fall during the rotarod test. (d) No significant difference was observed between sexes in the number of faults during the ladder beam task. (**e**–**f**) Male mice exhibited higher withdrawal threshold in the left hind foot and right hind foot during the Von Frey test. (**g**) No significant difference was observed between sexes in the response latency during the hot plate test.

In order to examine the sex difference in animal behavior, we subjected 8-week-old C57BL/6 male and female mice (n = 12) to a series of behavioral tests for a thorough investigation (Fig. 1a), to assess sensorimotor skills, emotional responses, and cognitive abilities. All tests were analyzed by post-hoc power test (Supplementary Table S1).

In the grip test, the average grip force of male mice was 89.72 ± 2.67 gf (gram force). This is significantly stronger than that of female mice, whose average grip strength was 78.61 ± 2.87 gf (t_(22)_ = 2.77,* p* = 0.01) (Fig. 1b). Rotarod and ladder beam tests were then performed to assess motor coordination. We detected no significant difference in either of these two tests, indicating comparable motor coordination between males and females (rotarod: t_(22)_ = 1.01, *p* = 0.32; ladder beam: t_(22) =_ 0.67, *p* = 0.50) (Fig. 1c and d).

We then assessed sensory sensitivity by measuring the response threshold to stimuli. In the Von Frey test, a filament was used to prick the plantar surface of a mouse’s hind feet with increasing force. The maximal force when the animal lifted their feet was then recorded as the withdrawal threshold. The withdrawal threshold of 4.24 ± 0.22 gf in the left hind feet was measured in males, while 3.42 ± 0.18 gf was measured in females (t_(22)_ = 2.884, *p* = 0.008) (Fig. 1e). For the right hind feet, the withdrawal threshold of 4.26 ± 0.12 gf was detected in males, with 3.60 ± 0.16 gf detected in female mice (t_(22)_ = 3.27, *p* = 0.003) (Fig. 1f). A sex difference was shown in the threshold for both the left and right feet, suggesting a lower tactile sensory sensitivity in male mice. The hot plate was another test for sensory sensitivity, and it recorded the latency of mice responding to thermal stimuli. No significant difference was observed in the response latency between sexes, implying similar sensitivity to thermal stimuli (U = 57.00, *p* = 0.40) (Fig. 1g). Taken together, a sex difference existed in grip and sensory sensitivity to tactile/touch, yet motor coordination and the sensitivity to heat was equivalent between male and female mice.

### No significant sex difference was detected in anxiety-like nor depressive-like behavior despite discrepancy in corticosterone levels

Divergence in anxiety-like behavior between male and female rodents has been discussed in previous studies. Interestingly, some studies have mentioned the difficulty of making conclusions since the outcomes differed in various tests, making interpretation rather challenging^4,9^.

**Figure 2:**
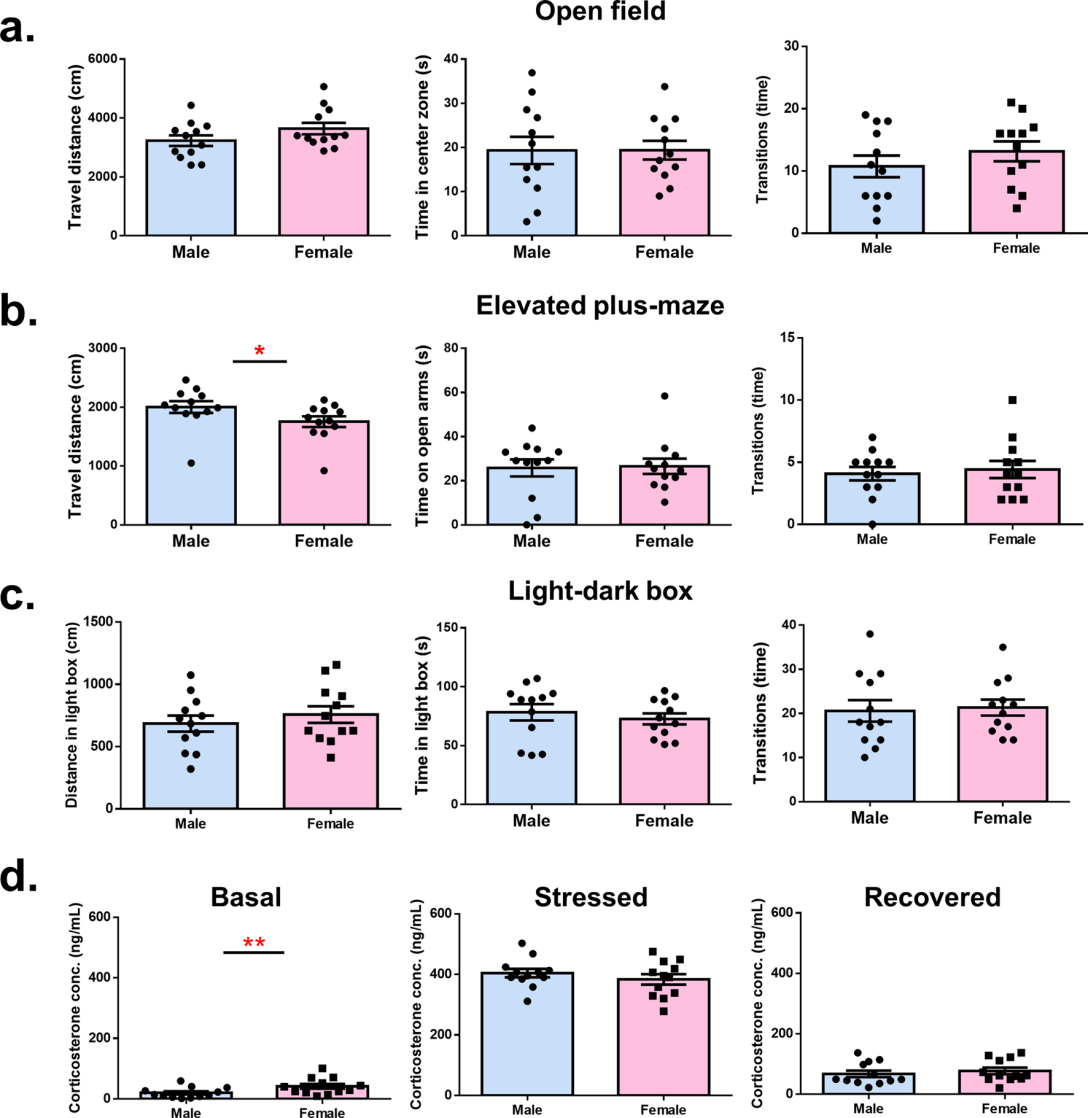
No significant sex difference was detected in anxiety-like behavior despite discrepancy in corticosterone levels. (**a**) In the open field test, no significant difference was measured between sexes in the travel distance, the time mice spent in the center zone, and the number of transitions. (**b**) In the elevated plus-maze, female mice exhibited longer travel distance, while the time mice spent on the open arms and the number of transitions were equivalent between the sexes. (**c**) In the light–dark box, no significant difference was measured between sexes in the travel distance in the light box, the time spent in the light box, and the number of transitions. (**d**) Female mice exhibited higher baseline plasma corticosterone level under basal conditions, while the plasma corticosterone levels were equivalent between sexes under stressed condition and under recovered condition.

To examine the sex effects on emotional responses, we tested for anxiety- and depression-like behavior in mice. Open field, elevated plus-maze, and light–dark box tests were conducted for the assessment of anxiety. In the open field test, both travel distance and the time mice spent in the center zone were comparable between the sexes, suggesting an equivalent level of locomotor activity and anxiety (distance: t_(22)_ = 1.54, *p* = 0.13; time: t_(22)_ = 0.01, *p* = 0.98) (Fig. 2a). In the elevated plus-maze, although female mice exhibited a longer travel distance as compared to males, there was no significant difference in the time spent on the open arms (distance: U = 31.00, *p* = 0.02; time: U = 55.00, *p* = 0.34) (Fig. 2b). We also calculated the relative exploration time on the open arms, which also showed equivalence between the groups (t_(22)_ = 1.10, *p* = 0.28), with males spending 38.62 ± 3.80% of exploration time on the open arms and females spending 42.90 ± 0.79% of exploration time. In the light–dark box, due to the inability to track the travel distance inside the dark box, we recorded the number of transitions across chambers instead. The number of transitions showed no difference between sexes, nor did the time spent in the light box (transition: t_(22)_ = 0.24, *p* = 0.80; time: t_(22)_ = 0.67, *p* = 0.50) (Fig. 2c). Together, the three anxiety-related behavioral tests showed good agreement that no apparent sexual difference was observed in these commonly used behavioral assays.

Despite no significant difference being observed in anxiety testing, we investigated whether plasma corticosterone concentrations differed between sexes. To test this, we quantified the plasma corticosterone levels at three different time points: the baseline condition (basal), after 30 min of restraint stress (stressed), and after 60 min of recovery from restraint stress (recovered). Under basal conditions, the concentration of plasma corticosterone was 20.05 ± 5.14 ng/ml in males and 41.60 ± 7.93 ng/ml in females (t_(22)_ = 0.03, *p* = 0.03) (Fig. 2d, left), indicating a higher baseline corticosterone level in female mice. After 30 min of restraint stress, the corticosterone levels increased to 350–450 ng/ml and there was no significant difference between sexes (t_(22)_ = 0.93, *p* = 0.35) (Fig. 2d, middle). One hour after the termination of the restraint stress, the corticosterone level decreased to 65–80 ng/ml, and no apparent difference was detected (t_(22)_ = 0.64, *p* = 0.52) (Fig. 2d, right). Overall, despite a comparable anxiety level exhibited in the behavioral assays, the baseline corticosterone level was shown higher in females than males; the corticosterone levels after restraint stress and after recovery from stress were otherwise equivalent between sexes.

**Figure 3:**
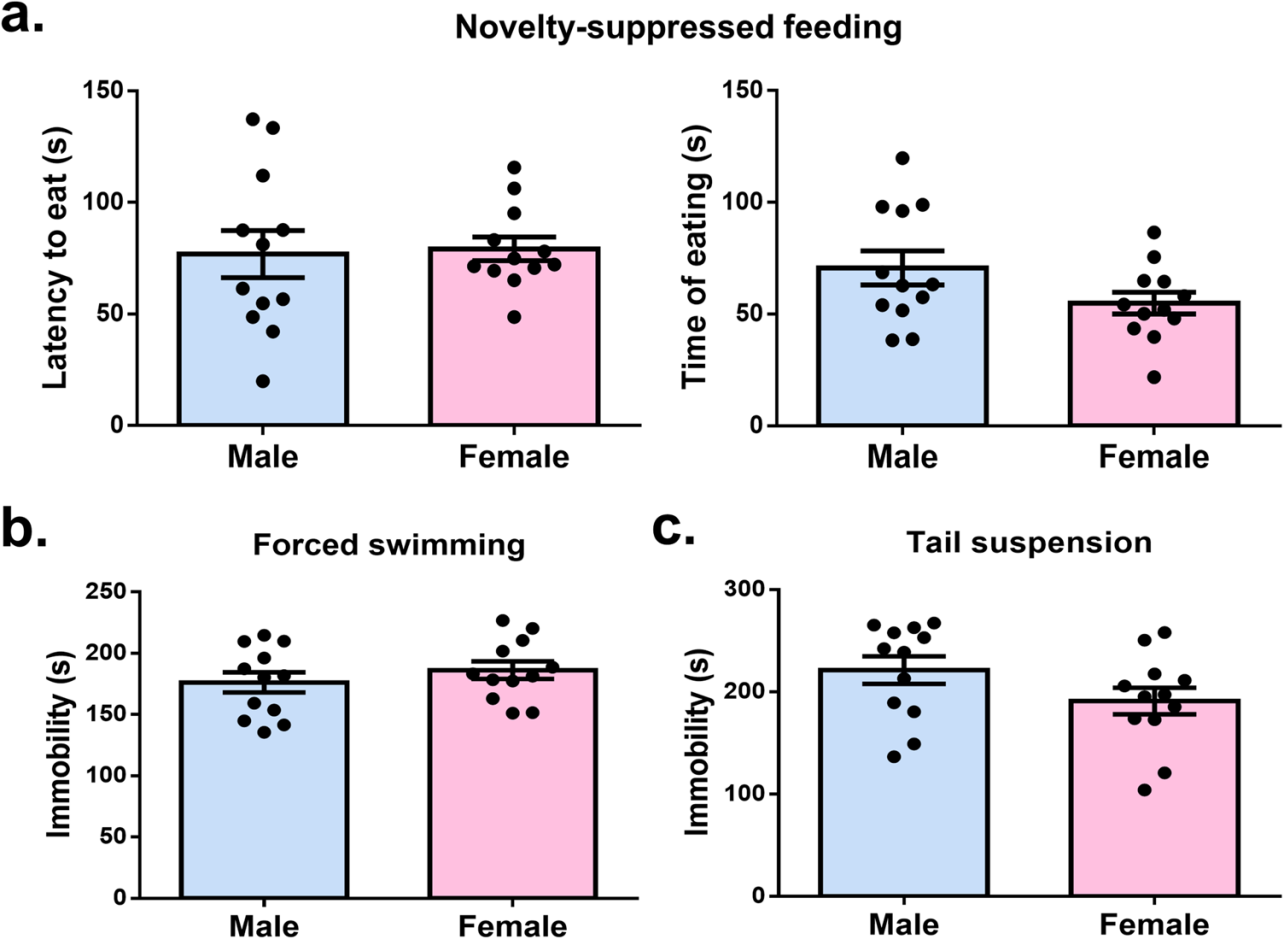
No significant sex difference was detected in depression-like behavior. (**a**) There was no significant difference between sexes in the latency to eat the food pellet nor the time of eating during the novelty suppressed feeding test. (**b**) There was no significant difference between sexes in the immobile time during the forced swimming test. (**c**) There was no significant difference between sexes in the immobile time during the tail suspension test.

For assessing depression-like behavior, novelty suppressed feeding, forced swimming, and tail suspension tests are commonly used. During the novelty suppressed feeding test, male and female mice exhibited a similar latency to start eating the food pellets and duration of eating, suggesting no significant difference in depression level (latency: t_(22)_ = 0.19, *p* = 0.84; time: t_(22)_ = 1.74, *p* = 0.09) (Fig. 3a). In the forced swimming test and tail suspension test, the immobility of mice was considered a depression-like behavior as mice stopped struggling under such stressed conditions. The time immobile was similar between males and females in both the forced swimming test and the tail suspension test (forced swimming: t_(22)_ = 0.91, *p* = 0.36; tail suspension: t_(22)_ = 1.61, *p* = 0.12) (Fig. 3b and c). Consequently, no significant sex difference was detected in depression-like behavior.

### No significant sex difference was detected in learning and memory

**Figure 4:**
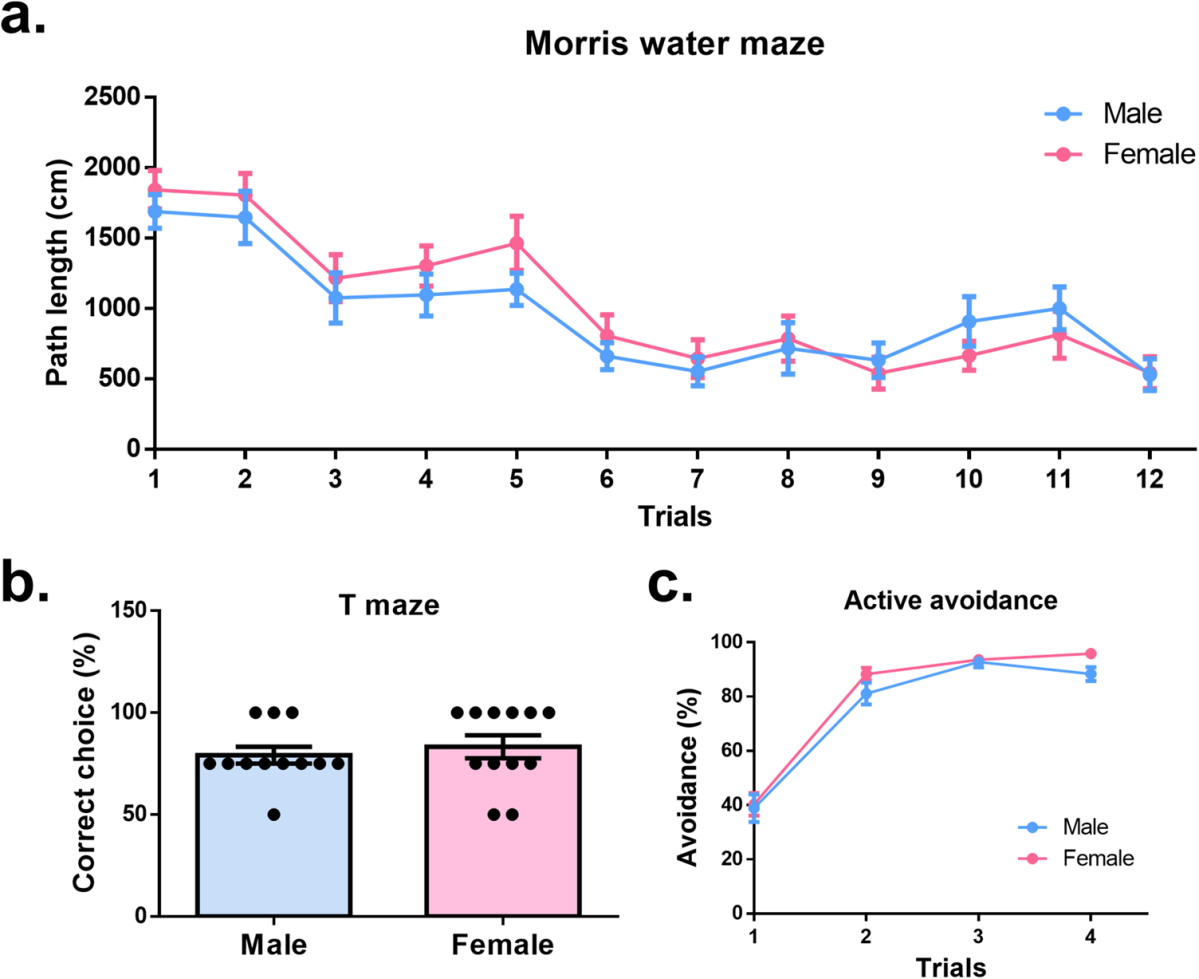
No significant sex difference was detected in learning and memory. (**a**) Males and females showed similar learning curve patterns in the Morris water maze test. (**b**) Males and females showed comparable percentage of correct choice in T maze. (**c**) Males and females showed similar learning curve patterns in the active avoidance test.

Studies have previously discussed sex differences in cognition, with some reporting sex to be a potential risk factor for cognitive deficits in neurodegenerative diseases^10–14^. To see whether a sex difference affects learning and memory, we conducted Morris water maze, T maze, and active avoidance tests. All these tests involve different aspects of cognitive function. The Morris water maze is one of the most widely used tests in evaluating spatial learning in rodents. With a platform hidden beneath the water surface, mice were trained to search for the platform. The path length mice required to reach the platform decreased with successive trials, showing mice became more adapt tat ascertaining the platform’s location. Mice displayed good learning curves in spatial learning and showing a significant effect of successive trials (F_(11,242)_ = 27.75, *p* < 0.0001). There was no remarkable difference between the male learning curve and the female one (F_(1,22)_ = 0.23, *p* = 0.63), indicating comparable spatial learning between sexes (Fig. 4a). The T maze is a test for assessing working memory, by which mice exploratory behavior and spatial discrimination are evaluated. The novel arm was defined as “correct” based on the assumption that a mouse would typically explore a new environment if it could distinguish it from an area it had previously explored. The results of the T maze tests showed an equivalent percentage of correct choices between males and females, implying similar working memory (U = 60.00, *p* = 0.46) (Fig. 4b). Active avoidance is a fear-motivated associated avoidance task, which requires the mice to associate the unconditioned stimuli (US) with conditioned stimuli (CS). The percentage of avoidance increased over time, revealing a significant effect of trials (F_(3,66)_ = 157.7, *p* < 0.0001). There was no evident difference between the two learning curves (F_(1,22)_ = 2.58, *p* = 0.12), showing comparable avoidance learning (Fig. 4c). In summary, no significant sex difference was found in cognitive behavioral testing.

### No significant sex difference was detected in adult hippocampal neurogenesis

**Figure 5:**
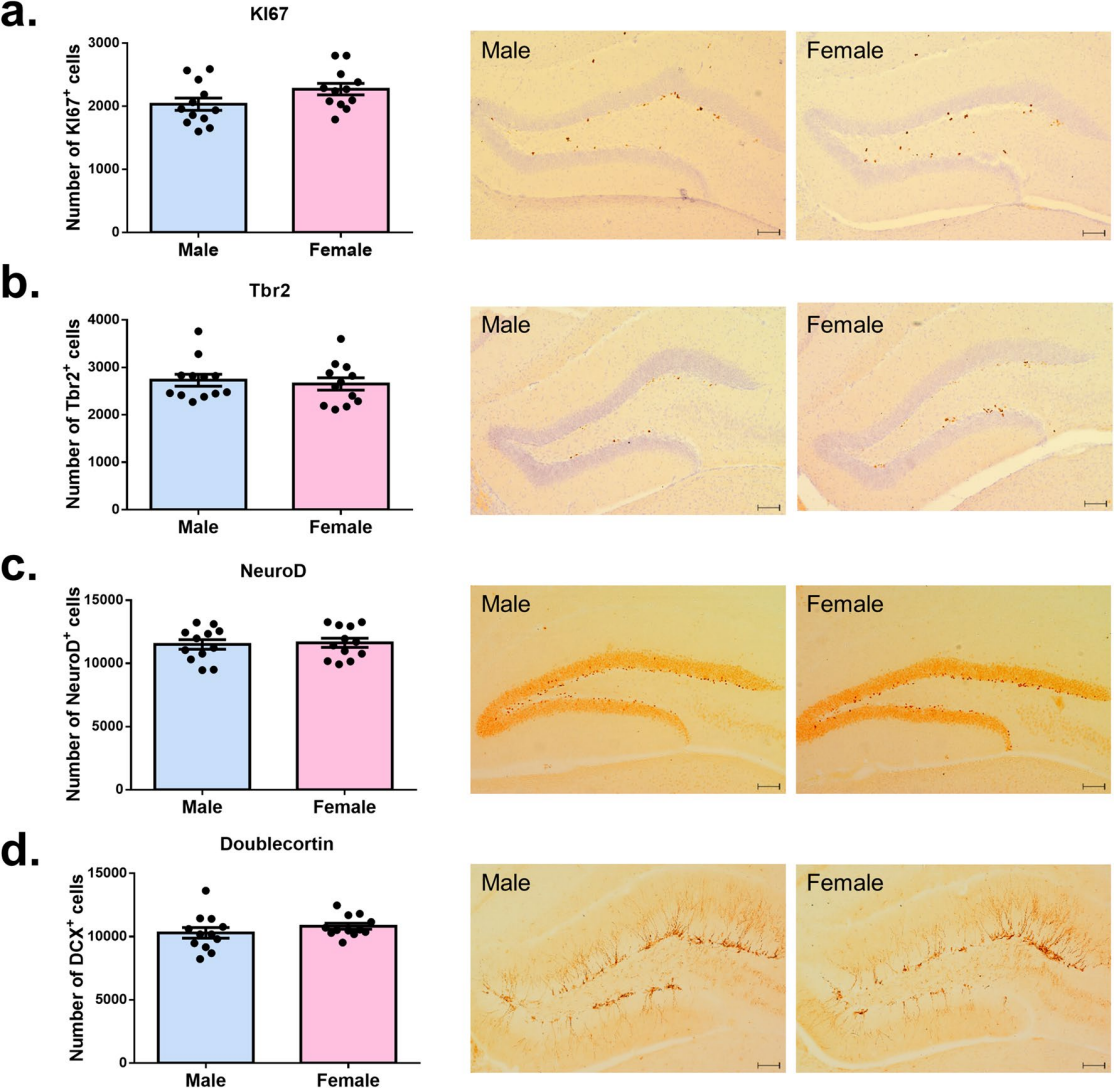
No significant sex difference was detected in adult hippocampal neurogenesis. (**a**) No significant difference between sexes was found in the KI67^+^ cells in the dentate gyrus. (**b**) No significant difference between sexes was found in the Tbr2^+^ cells in the dentate gyrus. (**c**) No significant difference between sexes was found in the NeuroD^+^ cells in the dentate gyrus. (**d**) No significant difference between sexes was found in the DCX^+^ cells in the dentate gyrus. Scale bars represent 100 µm.

Past research has revealed a sex difference in rat adult neurogenesis, suggesting that estrogen stimulates the number of newly born neurons in the rat hippocampus^8^. To test whether there is a sex difference in adult hippocampal neurogenesis in mice, we used immunochemical staining. KI67, Tbr2, NeuroD, and Doublecortin (DCX) were selected as targets for staining due to their distinct expression periods as neurogenesis markers. KI67 represents a marker for proliferating cells; Tbr2 is a marker for the early intermediate progenitors. NeuroD and DCX are markers for immature neurons^15^. We found no significant difference between males and females in the number of cells expressing neurogenesis markers (KI67: t_(22)_ = 1.77, *p* = 0.08; Tbr2: t_(22)_ = 0.43, *p* = 0.66; NeuroD: t_(22)_ = 0.25, *p* = 0.80; DCX: t_(22)_ = 1.07, *p* = 0.29), indicating that sex difference did not affect the adult hippocampal neurogenesis in mice (Fig. 5).

### No significant sex difference was detected in variance in overall tests

Traditionally, it has been considered that female subjects exhibited more variability than males due to the estrous cycle^16^. One study even demonstrated that female mice were less predictable near ovulation^17^. Nevertheless, there is no actual foundation for this belief, and recently increasing evidence has challenged this assumption^6,18^. To understand the effect of sex on variability, we compared the variances of male mice and females in the behavioral tests via a F-test (Table 1). Among 23 results of statistical analyses, only the latency to eat the food pellets in the novelty suppressed feeding test showed a significant difference in the variance, in which females exhibited a smaller variance than males. The comparison of variances showed an overall approximately equal variance between males and females.

**Table 1:** No significant sex difference was detected in variance in overall tests.

Tests	F_(11)_	*P* value	Tests	F_(11)_	*P* value
Grip	1.23	0.73	Light–dark box (duration)	2.21	0.20
Rotarod	1.38	0.59	Light–dark box (transition)	1.80	0.34
Ladder beam	1.31	0.66	CORT (basal)	2.38	0.16
Von Frey (L)	1.48	0.52	CORT (stressed)	1.49	0.51
Von Frey (R)	1.61	0.44	CORT (recovered)	1.02	0.96
Hot plate	1.34	0.63	Novelty suppressed feeding (latency)	3.90	0.03*
Open field (distance)	1.16	0.80	Novelty suppressed feeding (duration)	2.40	0.16
Open field (duration)	2.11	0.23	Forced swimming	1.31	0.65
Open field (transitions)	1.18	0.79	Tail suspension	1.06	0.92
Elevated plus maze (distance)	1.22	0.74	KI67	1.17	0.79
Elevated plus maze (duration)	1.25	0.70	Tbr2	1.08	0.90
Elevated plus maze (transitions)	1.62	0.44	NeuroD	1.07	0.90
Light–dark box (distance)	1.10	0.88	DCX	3.12	0.07

Variance of female mice was significantly smaller than that of male mice in the latency to eat the food pellet during the novelty suppressed feeding test (asterisk). All the other comparisons between the variance of males and females suggested no significant difference.

## Discussion

Animal behavioral testing is an important approach in biomedical research for assessing the effects of genes or drugs. Traditionally, male subjects are predominantly used to avoid any potential confounding factors coming from the female estrous cycle. Several studies have indicated that the estrous cycle in females could influence animal behavior and even adult neurogenesis in rodents^8,10,19,20^. It is also believed that females may exhibit higher variability than males^16,17^. Recently, it has been argued that female animals should be included in research to avoid sex biased results. Therefore, this study sought to examine whether sex difference influenced behavioral test results and variance in mice.

Our data shows females exhibited weaker grip strength and higher cutaneous sensory sensitivity. In contrast, there was no significant difference detected in other tests including motor coordination, anxiety-like behavior, depression-like behavior, and cognitive function. Overall, our data shows consistency with our own previous works that no significant sex difference was detected in animal behavior^21,22^. Nevertheless, some studies have reported different behavioral patterns between males and females in anxiety, learning, and memory^9,23^. Such contradictory results between different published studies could be attributed to many factors including the genetic backgrounds, environmental conditions, and handling procedure which we will discuss later^24–28^. Regarding the tests in which we detected sex differences, it was plausible that female mice exhibited weaker grip strength due to having significantly less skeletal muscle mass. A previous work also has demonstrated a similar result^29^. However, it is not fully understood why female mice displayed higher tactile sensitivity as compared to males. Different skin morphology might be a reason^30^.

When we tested the hypothalamus–pituitary–adrenal axis (HPA) reactivity, female mice exhibited a higher plasma corticosterone baseline compared to male mice, despite no significant differences in the corticosterone levels under the stressed nor recovered condition. It is not surprising that females exhibited a higher plasma corticosterone baseline since it has been demonstrated in other works^31–33^. Many studies have suggested that sex differences exist in the response of the HPA axis, directly or indirectly affected by gonadal steroid hormones, specific populations of corticotropin releasing factor (CRF) neurons in key stress-related brain regions, and the distribution of corticosteroid receptors in the brain^31,34–36^. As for the corticosterone level in the stressed condition, no difference between sexes was detected. There are two possibilities for this: one is that since plasma corticosterone increased dramatically after 30 min of stress, it is difficult to detect any potential small differences between males and females. The other one is that, since we only tested one time point, we cannot exclude the possibility that we missed the best timing to detect the sex difference. For the corticosterone level after one hour of recovery, theoretically the result should be similar to the baseline. A longer period of time, or a time course, may be required to see the changes of plasma corticosterone after recovery, and any potential sex difference.

Besides behavioral phenotypes, we also compared the within group variations between males and females. Traditionally, it is believed that estrous cycle increases the variability in female subjects^16,17^. However, our results suggested that males and females displayed similar within group variations. This is in agreement with other studies and meta-analysis which support the idea that female sex or the endogenous estradiol level does not exert such a profound effect on variability^18,37–40^. Based on this finding, we believe that female subjects should, justifiably, be included in future scientific research.

Moreover, we measured adult neurogenesis, which occurs in the dentate gyrus of the hippocampus. Past research has revealed an estrogen-enhanced cell proliferation in female rats during the proestrus phase^8^. However, Lagace et al.^41^ found that the estrous cycle does not affect the adult neurogenesis in mice, suggesting the discrepancy between rats and mice. In our study, we used several markers for proliferating cells and differentiating neurons, and we failed to detect any significant difference between male and female mice. It is likely that the estrogen-enhanced cell proliferation in rats cannot be applied to mice; some further investigation is required to explore the influence of estrogen on mouse adult neurogenesis.

Nonetheless, there were limitations and flaws in the experimental design, which might affect the results to different degrees. First, because of the limited sample size, many of our results were underpowered. We understand this is less than ideal since low power could increase the probability of reporting a false result. However, low power has long been a problem in animal studies, whose median statistics power is between ~ 8% and ~ 31% in neuroscience^26,42^. It is not a feasible solution to reach acceptable power by increasing the sample size, with very small difference in effect size potentially requiring a great number of animals and this posing both logistical and ethical challenges. Still, it is possible that sex difference plays a role with larger effect sizes. Second, we used a fairly busy schedule for the behavioral testing with limited rest between tests. This might cause carryover effect or other potential interference on mouse performance, becoming confounding factors during the assessment of animal behavior. Third, the subjects we used in this study were C57BL/6 mice, and no other strains nor treatments were tested. Our results can only represent for the C57BL/6 strain under basal conditions since animals’ responses could differ with genetic backgrounds and environmental factors. There are potential interactions between sex, genes, and/or drugs. This also brings a challenge to the reproducibility of our results^43^.

In conclusion, overall not significant effects of sex difference were shown in our behavior tests, along with the within group variation, at least with the sample size we used in this project. Arguably to avoid sex biased data, it is important to take this into account and consider experimental design protocols that utilize both male and female animals so any such interactions can be detected and explored. In summary, this study provides evidence that there was no significant sex difference in the commonly used behavioral assays under basal conditions in mice. We suggest that consideration is given to female subjects being included in experimental design.

## Methods

### Animals

Experiments were performed on 12 male and 12 female C57BL/6 mice, ordered from National Laboratory Animal Center, Taiwan. They were housed in AAALAC certified animal facility in a 12:12 h light dark cycle (7:00 light on, 19:00 light off) at a temperature of 22 °C and a humidity level of 60–70%. Animals had ad libitum access to food and water. All mice were raised in the same housing room, with both males and females were group-housed, four in one cage, in the individually ventilated cage (IVC) system. In total there were 3 cages of male mice and 3 cages of females. No fighting nor fur loss was observed. All procedures were carried out in accordance with the local regulations and approved by Institutional Animal Care and Use Committee at Chang Gung University (Permit Number: CGU107-025). Zoletil was used prior to dislocation when sacrificing mice and tissue harvesting.

### Behavioral testing

Mice were subjected to behavioral tests at the age of 8–12 weeks as shown in the timeline featured in Fig. 1a. All behavioral tests were conducted during the mouse active phase. The experimental details were described below and in previous studies^21,22^. For the light intensity, we conducted the open field under a lamp, whose light intensity was 400 lx. For elevated plus-maze and novelty-suppressed feeding, we used the yellow light whose intensity was 350 lx. As for the other behavioral tests, it was conducted in the behavioral room under white light, where the light intensity was 440 lx. All mice were numbered from M1 to M12 (male) or F1 to F12 (female). In every behavioral assay, mice were tested in order of their designed numbers, and the sexes were also alternately ordered. Due to overt differences in the animal appearance between males and females, the experimenter was not blind to the animal sex during the behavioral testing. However, most of the statistical data were automatically detected and measured by the computer.

#### Grip

The manufacturer's instructions for the grip strength meter (47,200, UGO BASILE) were followed. The grasping bar was fitted to a forced transducer that connected to the peak amplifier. Mice were allowed to practice gripping three times before the formal test. Then they were pulled by the tail by the researcher. The grip force when mice lost hold of the grasping bar was recorded as the maximal grip force. Each mouse was tested three times and the average grip force was recorded.

#### Rotarod

Rotarod was used to assess mouse motor coordination^44^. Mice were placed on the rotarod (Ugo BASILE), where the rod rotated at an initial speed of 4 rpm and an acceleration speed of 9 rpm per minute. Once acceleration had been triggered, the time taken for mice to fall off the rod was recorded.

#### Ladder beam (Ladder rung walking test)

Ladder beam was used to assess mouse motor coordination^45^. Mice were placed at one end of the apparatus, and their home cage was placed at the other end. Animals were allowed to practice crossing the ladder beam apparatus once. During the formal tests, 20 metal rungs of the apparatus were removed and the number of times mice missed a rung when crossing was recorded. Each mouse crossed the ladder twice and the average number of faults (missed rungs) was calculated.

#### Von frey

Von Frey was used to test for the tactile sensory sensitivity^46^. Mice were placed individually in an elevated acrylic chamber equipped with a wire mesh floor, and a Von Frey filament (BiosebLab) was applied from underneath, pricking the hind paws. The strength of Von Frey filament would continuously increase until paw withdrawal, and the withdrawal threshold was recorded. Each mouse was pricked for five times both on the right hind paw and the left hind paw. The average withdrawal threshold of each hind paw was recorded.

#### Hot plate

Hot plate was used to test for the thermal sensory sensitivity^47^. Mice were placed on the hot plate (Socrel DS37) which maintained the temperature at 55 °C. The time taken for the mice to jump (defined as lifting four limbs at the same time) was recorded.

#### Open field

Open field was used to assess the anxiety level^48,49^. Mice were at liberty to explore a circular-shaped area (diameter = 60 cm) for 5 min with a lamp being the only light source illuminating the center zone. The travel distance, time spent in the center zone (diameter = 40 cm), and frequency of entering the center zone were recorded by Ethovision software. As mice avoided the center zone, the decreased exploration (increased thigmotaxis) was interpreted as higher anxiety.

#### Elevated-Plus maze

Elevated plus-maze was used to assess the anxiety level^50^. It consisted of two open arms (30 × 5 cm) and two enclosed arms which featured 15 cm-high walls. The maze was elevated 40 cm above the floor. Mice were placed in the maze for 5 min. The travel distance, time spent in the open arms, and frequency of entering the open arms were recorded using the Ethovision software. As mice avoided the open arms, the decreased exploration was interpreted as higher anxiety.

#### Light–dark box

Light–dark box was used to assess the anxiety level^51^. It was divided into a covered dark box and a coverless light box. At the beginning of the test, mice were put into the covered dark box and allowed to transit freely between the dark and coverless light box via a door. Mice were at liberty to move in the chamber for 5 min under white light. The travel distance and duration in the light box were recorded by Ethovision software along with the frequency of entering the light box. As mice avoided the light box, the decreased exploration was interpreted as higher anxiety.

#### Novelty suppressed feeding

Novelty suppressed feeding was used to assess the depression-like behavior^52,53^. Mice were prevented from consuming food for 24 h before testing. During the test, mice were placed in the center of a standard cage, and allowed to move freely. The cage featured food pellets placed in the four corners and a lamp illuminating the center zone. The latency to approach the food and the time spent on eating within 5 min were recorded manually. The longer latency time before the mice ate the food and the measured decrease in eating time were considered as indicators of depression.

#### Forced swimming test

Forced swimming test was used to assess the depression-like behavior^54^. Mice were placed into a plastic cylinder (diameter = 20 cm, height = 50 cm) which was filled with water at room temperature (22℃), up to a height of 30 cm, and the behavior of mice was recorded for 6 min using the EthoVision software. The increased immobility was considered as an indicator of depression.

#### Tail suspension test

Tail suspension test was used to assess the depression-like behavior^55^. Mice were suspended by their tail at a height of 20 cm elevated above the floor and their behavior was recorded for 6 min using the EthoVision software. The increased immobility was considered as an indicator of depression.

#### Morris water maze

Morris water maze was used to evaluate the spatial learning and memory^56^. A circular water tank (diameter = 130 cm) was filled with water at 22℃ that was colored with white acrylic turpentine, and a circular platform (diameter = 20 cm) was submerged 1 cm below the water surface. Mice were put into the tank at three different starting positions; each of the three quadrants other than the quadrant with the platform. Each mouse was trained to search for the hidden platform and stay on it for 30 s, three trials a day for four consecutive days. The time each mouse took to find the platform was recorded using EthoVision software. The decreased distance needed to reach the platform was a measure of spatial learning.

#### T maze

T maze was used to evaluate the working memory^57^. All guillotine doors in the T-maze were lifted before the test. A mouse was then placed in the start area and allowed to choose a goal arm. The mouse was confined to the chosen arm and start area for 30 s by quietly sliding the other door down. The mouse was then removed and immediately placed back into the maze to select an arm. The correct choice was defined as the mouse exploring the novel arm. Each mouse repeated the experiment three times, and the average percentage of correct choices was calculated. As mice recognized the familiar arm which they just explored, the percentage of correct choices was indicative of working memory.

#### Active avoidance

Active avoidance was used to evaluate the avoidance learning which involved the fear-conditioning^58^. Mice were placed in a two-compartment shuttle box equipped with a speaker and a light bulb in each compartment (Med Associates, Inc). Conditioned stimuli (5 s of light and 8 kHz, 85 dB tone) were given in the compartment where the mouse was located, followed by an unconditioned stimulus of an electric footshock (0.3 mA) from underlying conducting rods. Once the mice moved to the other compartment or the cut-off time (10 s) was up, the conditioned stimuli ceased. After a random inter-session interval (range 3–10 s), the next session started. Mice repeated 50 sessions a day for 4 consecutive days. The increased percentage of avoidance was an indicator of learning.

### Immunohistochemistry

Dissected brains were fixed in 4% paraformaldehyde overnight followed by dehydration in 25% sucrose. Brain sections at a thickness of 40 µm were collected and stored in anti-freeze buffer before staining.

For immunohistochemical staining, brain sections were mounted on SuperFrost Plus slides (Thermo) and air-dried overnight. The slides were incubated with 0.01 M citric acid buffer (pH = 6.4) for 20 min at 95 °C (40 min in the case of KI67), 3% H_2_O_2_ for 5 min, and then diluted primary antibody (KI67 1:2500 Abcam; Tbr2 1:1000 Abcam; NeuroD 1:500 Santa Cruz; DCX 1:3000 Abcam) at room temperature overnight; slides were washed with PBS for 15 min (3 times) between each step. Subsequently, we used a standard IgG ABC kit (Vector Lab) according to the manufacturer’s instructions and incubated the slides for 5–10 min with 3,3’-Diaminobenzidine (DAB) tablets (Sigma). Sections were then counterstained with cresyl violet and mounted in a DPX mountant (Sigma).

For quantification, all sections were examined under a microscope with a magnification of 200x. Antibody-stained cells were counted on the dentate gyrus bilaterally every eight sections through the entire extent of the granule cell layer (six sections per animal). The number of cell counts was then multiplied by eight to obtain an estimate of total antibody-stained cells in the dentate gyrus (DG).

### Corticosterone assay

We collected the blood samples by facial puncture under three different states: basal, stressed, and recovered. The basal blood was collected at 21:00, while the stressed and recovered blood were collected at 21:30 and 22:30, right after 30 min of stress and 1 h of recovery, respectively. The blood samples of different states were collected on different days, avoiding the potential interference. Plasma was separated from whole blood by centrifugation (3000 rpm, 4 °C, 15 min) and stored at  − 80 °C until used. Plasma corticosterone concentration was measured using the Corticosterone ELISA kit (ADI-900-097, Enzo Life Sciences) according to the manufacturer’s instructions. The chosen assay for mouse corticosterone analysis has been validated in our lab, which we obtain consistent values from different batches of control mice and others’ results^22,59–61^. The intra-assay coefficients of variation (CV) for our test was 4.83%, and the inter-assay CV was 10.34%, which were both acceptable for ELISA results.

### Statistical analysis

The data were presented as mean ± SEM for each group. *p < 0.05, **p < 0.01, ***p < 0.001. All results were statistically analyzed for normal distribution by D’Agostino and Pearson omnibus normality test using IBM SPSS software and were performed using Graphpad Prism software. Normally distributed data were analyzed via an analysis of variance (ANOVA) and unpaired t-test as appropriate; non-normal data were analyzed via Mann–Whitney test. The comparison of variance was analyzed using F-test.

### Supplementary Information

Supplementary Table S1.

## Data Availability

The datasets generated and analysed during this study are available from the corresponding author on reasonable request.

## References

[1] Ericsson AC, Crim MJ, Franklin CL (2013). A brief history of animal modeling. Mo. Med..

[2] Perlman RL (2016). Mouse models of human disease: An evolutionary perspective. Evol., Med., Public Health.

[3] Levine S (1966). Sex differences in the brain. Sci. Am..

[4] Johnston AL, File SE (1991). Sex differences in animal tests of anxiety. Physiol. Behav..

[5] Kokras N, Dalla C (2014). Sex differences in animal models of psychiatric disorders. Br. J. Pharmacol..

[6] Beery AK, Zucker I (2011). Sex bias in neuroscience and biomedical research. Neurosci. Biobehav. Rev..

[7] Solberg LC (2006). A protocol for high-throughput phenotyping, suitable for quantitative trait analysis in mice. Mamm. Genome.

[8] Tanapat P, Hastings NB, Reeves AJ, Gould E (1999). Estrogen stimulates a transient increase in the number of new neurons in the dentate gyrus of the adult female rat. J. Neurosci..

[9] Scholl JL, Afzal A, Fox LC, Watt MJ, Forster GL (2019). Sex differences in anxiety-like behaviors in rats. Physiol. Behav..

[10] Yagi S, Galea LAM (2019). Sex differences in hippocampal cognition and neurogenesis. Neuropsychopharmacology.

[11] Brookmeyer R, Gray S, Kawas C (1998). Projections of Alzheimer's disease in the United States and the public health impact of delaying disease onset. Am. J. Public Health.

[12] McPherson S, Back C, Buckwalter JG, Cummings JL (1999). Gender-related cognitive deficits in Alzheimer's disease. Int. Psychogeriatr..

[13] Han M (2012). Gender differences in cognitive function of patients with chronic schizophrenia. Prog. Neuro-psychopharmacol. Biol. Psychiatry.

[14] Leung MD, Psych CMRC (2000). Sex differences in schizophrenia, a review of the literature. Acta Psychiatrica Scandinavica.

[15] Yeh CY, Wu KY, Huang GJ, Verkhratsky A (2023). Radial stem astrocytes (aka neural stem cells): identity, development, physio-pathology and therapeutic potential. Acta Physiol..

[16] Chari T, Griswold S, Andrews NA, Fagiolini M (2020). The stage of the estrus cycle is critical for interpretation of female mouse social interaction behavior. Front. Behav. Neurosci..

[17] Kästner N, Richter SH, Gamer M, Kaiser S, Sachser N (2017). What a difference a day makes—female behaviour is less predictable near ovulation. R. Soc. Open Sci..

[18] Beery AK (2018). Inclusion of females does not increase variability in rodent research studies. Curr. Opin. Behav. Sci..

[19] Williams CL, Barnett AM, Meck WH (1990). Organizational effects of early gonadal secretions on sexual differentiation in spatial memory. Behav. Neurosci..

[20] Grissom EM, Hawley WR, Hodges KS, Fawcett-Patel JM, Dohanich GP (2013). Biological sex influences learning strategy preference and muscarinic receptor binding in specific brain regions of prepubertal rats. Hippocampus.

[21] Chen YJ (2021). Follistatin mediates learning and synaptic plasticity via regulation of Asic4 expression in the hippocampus. Proc. Natl. Acad. Sci. U S A.

[22] Tsao CH, Flint J, Huang GJ (2022). Influence of diurnal phase on behavioral tests of sensorimotor performance, anxiety, learning and memory in mice. Sci. Rep..

[23] Chen CS, Knep E, Han A, Ebitz RB, Grissom NM (2021). Sex differences in learning from exploration. Elife.

[24] Jonasson Z (2005). Meta-analysis of sex differences in rodent models of learning and memory: A review of behavioral and biological data. Neurosci. Biobehav. Rev..

[25] Shoji H, Miyakawa T (2019). Age-related behavioral changes from young to old age in male mice of a C57 BL/6J strain maintained under a genetic stability program. Neuropsychopharmacology reports.

[26] Button KS (2013). Power failure: why small sample size undermines the reliability of neuroscience. Nat. Rev. Neurosci..

[27] Sorge RE (2014). Olfactory exposure to males, including men, causes stress and related analgesia in rodents. Nat. Methods.

[28] Balcombe JP, Barnard ND, Sandusky C (2004). Laboratory routines cause animal stress. J. Am. Assoc. Lab. Anim. Sci..

[29] Azzi L, El-Alfy M, Martel C, Labrie F (2005). Gender differences in mouse skin morphology and specific effects of sex steroids and dehydroepiandrosterone. J. Investig. Dermatol..

[30] Ross JL, Queme LF, Lamb JE, Green KJ, Jankowski MP (2018). Sex differences in primary muscle afferent sensitization following ischemia and reperfusion injury. Biol. Sex Differ..

[31] Oyola MG, Handa RJ (2017). Hypothalamic–pituitary–adrenal and hypothalamic–pituitary–gonadal axes: Sex differences in regulation of stress responsivity. Stress.

[32] Nguyen K, Kanamori K, Shin CS, Hamid A, Lutfy K (2020). The impact of sex on changes in plasma corticosterone and cotinine levels induced by nicotine in c57bl/6j mice. Brain sciences.

[33] Bethin KE, Vogt SK, Muglia LJ (2000). Interleukin-6 is an essential, corticotropin-releasing hormone-independent stimulator of the adrenal axis during immune system activation. Proc. Natl. Acad. Sci..

[34] Handa RJ, Burgess LH, Kerr JE, O'Keefe JA (1994). Gonadal steroid hormone receptors and sex differences in the hypothalamo-pituitary-adrenal axis. Horm. Behav..

[35] Babb JA, Masini CV, Day HE, Campeau S (2013). Sex differences in activated corticotropin-releasing factor neurons within stress-related neurocircuitry and hypothalamic–pituitary–adrenocortical axis hormones following restraint in rats. Neuroscience.

[36] MacLusky NJ, Yuan H, Elliott J, Brown TJ (1996). Sex differences in corticosteroid binding in the rat brain: An in vitro autoradiographic study. Brain Res..

[37] Mogil JS, Chanda ML (2005). The case for the inclusion of female subjects in basic science studies of pain. Pain.

[38] Levy DR (2023). Mouse spontaneous behavior reflects individual variation rather than estrous state. Curr. Biol..

[39] Prendergast BJ, Onishi KG, Zucker I (2014). Female mice liberated for inclusion in neuroscience and biomedical research. Neurosci. Biobehav. Rev..

[40] Kaluve AM, Le JT, Graham BM (2022). Female rodents are not more variable than male rodents: A meta-analysis of preclinical studies of fear and anxiety. Neurosci. Biobehav. Rev..

[41] Lagace DC, Fischer SJ, Eisch AJ (2007). Gender and endogenous levels of estradiol do not influence adult hippocampal neurogenesis in mice. Hippocampus.

[42] Bonapersona V, Hoijtink H, Consortium R, Sarabdjitsingh RA, Joels M (2021). Increasing the statistical power of animal experiments with historical control data. Nat Neurosci.

[43] Voelkl B (2020). Reproducibility of animal research in light of biological variation. Nat. Rev. Neurosci..

[44] Hamm RJ, Pike BR, O'DEL DM, Lyeth BG, Jenkins LW (1994). The rotarod test: an evaluation of its effectiveness in assessing motor deficits following traumatic brain injury. J. Neurotrauma.

[45] Metz GA, Whishaw IQ (2002). Cortical and subcortical lesions impair skilled walking in the ladder rung walking test: a new task to evaluate fore-and hindlimb stepping, placing, and co-ordination. J. Neurosci. Methods.

[46] Carter M, Shieh JC, Nociception. (2010). Guide to research techniques in neuroscience.

[47] Eddy NB, Leimbach D (1953). Synthetic analgesics. II. Dithienylbutenyl-and dithienylbutylamines. J. Pharmacol. Exp. Therapeutics.

[48] Hall, C. & Ballachey, E.L. A study of the rat's behavior in a field. A contribution to method in comparative psychology. *University of California Publications in Psychology* (1932).

[49] Denenberg VH (1969). Open-field behavior in the rat: What does it mean?. Ann. New York Acad. Sci..

[50] Pellow S, Chopin P, File SE, Briley M (1985). Validation of open: closed arm entries in an elevated plus-maze as a measure of anxiety in the rat. J. Neurosci. Methods.

[51] Crawley J, Goodwin FK (1980). Preliminary report of a simple animal behavior model for the anxiolytic effects of benzodiazepines. Pharmacol. Biochem. Behav..

[52] Bodnoff SR, Suranyi-Cadotte B, Aitken DH, Quirion R, Meaney MJ (1988). The effects of chronic antidepressant treatment in an animal model of anxiety. Psychopharmacology.

[53] Dulawa SC, Hen R (2005). Recent advances in animal models of chronic antidepressant effects: the novelty-induced hypophagia test. Neurosci. Biobehav. Rev..

[54] Porsolt R, Bertin A, Jalfre M (1977). Behavioral despair in mice: a primary screening test for antidepressants. Arch. Int. Pharmacodyn. Ther..

[55] Steru L, Chermat R, Thierry B, Simon P (1985). The tail suspension test: a new method for screening antidepressants in mice. Psychopharmacology.

[56] Morris R (1984). Developments of a water-maze procedure for studying spatial learning in the rat. J. Neurosci. Methods.

[57] Deacon RM, Rawlins JNP (2006). T-maze alternation in the rodent. Nat. Protocols.

[58] Mowrer OH, Lamoreaux RR (1946). Fear as an intervening variable in avoidance conditioning. J. Comp. Psychol..

[59] Chang S (2018). NPTX2 is a key component in the regulation of anxiety. Neuropsychopharmacology.

[60] Hare BD, Beierle JA, Toufexis DJ, Hammack SE, Falls WA (2014). Exercise-associated changes in the corticosterone response to acute restraint stress: evidence for increased adrenal sensitivity and reduced corticosterone response duration. Neuropsychopharmacology.

[61] McClennen SJ, Cortright DN, Seasholtz AF (1998). Regulation of pituitary corticotropin-releasing hormone-binding protein messenger ribonucleic acid levels by restraint stress and adrenalectomy. Endocrinology.

