# Supplementary material for: The influence of sex difference on behavior and adult hippocampal neurogenesis in C57BL/6 mice
Source: Sci Rep. 2023 Oct 12;13:17297. doi: 10.1038/s41598-023-44360-8 (PMC10570284; doi:10.1038/s41598-023-44360-8)
Supplement: Supplementary file 1 — Supplementary Table S1. [file 41598_2023_44360_MOESM1_ESM.docx]

**Supplementary Table S1.**

| **Tests** | **Power (%)** | **Tests** | **Power (%)** |
| --- | --- | --- | --- |
| **Grip** | 75.6 | **Light-dark box (duration)** | 9.9 |
| **Rotarod** | 16.3 | **Light-dark box (transition)** | 5.6 |
| **Ladder beam** | 9.9 | **CORT (basal)** | 58.7 |
| **Von Frey (L)** | 78.7 | **CORT (stressed)** | 14.6 |
| **Von Frey (R)** | 88.0 | **CORT (recovered)** | 9.4 |
| **Hot plate** | 8.1 | **Novelty suppressed feeding (latency)** | 5.4 |
| **Open field (distance)** | 31.6 | **Novelty suppressed feeding (duration)** | 38.6 |
| **Open field (duration)** | 5.0 | **Forced swimming** | 14.2 |
| **Open field (transitions)** | 16.6 | **Tail suspension** | 33.7 |
| **Elevated plus maze (distance)** | 41.5 | **KI67** | 39.7 |
| **Elevated plus maze (duration)** | 5.2 | **Tbr2** | 7 |
| **Elevated plus maze (transitions)** | 6.5 | **NeuroD** | 5.7 |
| **Light-dark box (distance)** | 11.6 | **DCX** | 17.7 |

**Table S1. Statistic power of all behavioral tests.**

Statistic power of all behavioral tests, analyzed by post-hoc power analysis.
